# Treatment for the Thoracic Ossification of the Posterior Longitudinal Ligament and Ossification of the Ligamentum Flavum

**DOI:** 10.3390/jcm11164690

**Published:** 2022-08-11

**Authors:** Masaaki Machino, Kenichiro Sakai, Toshitaka Yoshii, Takeo Furuya, Sadayuki Ito, Naoki Segi, Jun Ouchida, Shiro Imagama, Hiroaki Nakashima

**Affiliations:** 1 Department of Orthopedic Surgery, Nagoya University Graduate School of Medicine, 65 Tsurumaicho, Showa Ward, Nagoya 466-8550, Japan; 2 Department of Orthopedic Surgery, Saiseikai Kawaguchi General Hospital, 5-11-5 Nishikawaguchi, Kawaguchishi 332-8558, Japan; 3 Department of Orthopedic Surgery, Tokyo Medical and Dental University, 1-5-45 Yushima, Bunkyo Ward, Tokyo 113-8519, Japan; 4 Department of Orthopedic Surgery, Chiba University Graduate School of Medicine, 1-8-1 Inohana, Chuo Ward, Chiba 260-8670, Japan

## 1. Thoracic Ossification of the Posterior Longitudinal Ligament (T-OPLL)

The T-OPLL natural course has not been extensively reported, and evidence to support the timing of surgery is also lacking. Owing to the correlation between symptom duration and a low postoperative improvement rate, which has been described, the choice to perform surgery should be proactively examined in patients with progressing myelopathy [1].

In theory, direct decompression induced by OPLL appears plausible. However, operations near the spinal cord that cause extensive injury increase the risk of exacerbating neurological disorders. Therefore, techniques for posterior surgery for T-OPLL without ossification excision are frequently selected. Posterior procedures are divided into posterior decompression alone (laminectomy and laminoplasty) and posterior decompression and fusion (including the method combining fusion with laminectomy and laminoplasty and a kyphosis-reducing procedure (dekyphosis)). Both increased instability induced by posterior invasion into the tissue supporting the spinal column and advanced kyphosis due to age-related intervertebral degeneration and the concurrent compression fracture of the spine exacerbate neurological impairment in posterior decompression alone. Except in individuals with continuous ossification, decompression alone for the mid-thoracic spine is rarely used. Fusion is unnecessary when performing posterior surgery for T-OPLL. Notably, surgical outcomes are good with posterior decompression alone for OPLL when the severity is at a level at which alignment indicates mild lordosis and kyphosis, such as in the upper thoracic spine [1].

The recent increase in improvement rate has been affected by simultaneous fusion via the posterior approach, and various procedures, including posterior decompression and fusion, anterior decompression and fusion, and anterior decompression via the posterior approach, are useful surgical procedures [1]. However, a high incidence of complications following T-OPLL surgery has been reported; thus, further research is required to assess the benefits while evaluating symptom improvement and complication risks [1].

Additionally, factors related to inadequate long-term improvement after surgery include symptom duration, multilevel decompression, cervical OPLL concurrent with the ossification of the ligamentum flavum (OLF), maximum ossification located at the T1–T4 levels, length of the long axis of the OPLL, ossification occupancy rate within the spinal canal, decompression of five or more vertebral bodies, insufficient kyphosis correction during posterior decompression and fixation, intraoperative neurophysiological monitoring (IONM) aggravation, simultaneous surgery of the cervical and lumbar spine, instrumentation utilization, and mid-thoracic spine position [2].

According to the evaluation of symptoms such as numbness or spasticity in a persistent prone or supine posture, changes in spinal alignment while in bed worsened the symptoms. This test was deemed negative in cases where symptoms did not progress after five minutes because these symptoms worsen within one minute in most cases. The authors referred to this test as a prone and supine position test (PST), and situations when symptoms worsened were considered positive [3].

Preoperative physical examinations of PST results are significant and independent elements that affect the success of surgery. Although the PST is positive, the result might not be favorable, even if T-OPLL patients can walk with assistance. Because these patients’ symptoms worsen while in bed and their spinal cord alignment changes somewhat, PST can identify fragile spinal cord damage before surgery. Preoperative PST evaluation is recommended in all T-OPLL cases to identify any serious spinal cord damage and to allow the planning of surgery with appropriate timing to improve the likelihood of a successful procedure [3].

Imagama S et al. prospectively determined the surgical strategy for beak-type T-OPLL, with the additional resection of T-OPLL from a posterior approach planned for three weeks after the first surgery if the patient shows no improvement or has worsening symptoms [4]. The initial procedure involved posterior decompression and dekyphotic corrective fusion with instrumentation. The ineffectiveness of posterior decompression and fusion with instrumentation was found to be associated with preoperative severe motor paralysis, immobility, positive PST, radiographic spinal cord compression with beak-type T-OPLL, intraoperative residual spinal cord compression, and IONM aggravation. Their two-stage strategy may suit beak-type T-OPLL surgery. In addition, they discovered that resection at an anterior site of the spinal cord from a posterior approach (RASPA) surgery from a posterior approach has the advantage of additional dekyphosis after the most prominent beak-type T-OPLL resection, which enables additional spinal cord decompression without the complete resection of multilevel beak-type T-OPLL and reduces invasiveness [4].

The perioperative complications of T-OPLL surgery were assessed in a prospective, multicenter, and national study [5]. The incidence of postoperative complications was 51.3%, and the most frequent one was increased motor strength in the lower extremities (32.2%). The study’s five different types of surgeries did not significantly differ from one another’s 1-year surgical results, indicating that each case’s surgical treatment was selected appropriately. Most cases (74%) had posterior decompression and fusion with instrumentation, with or without concomitant dekyphosis, and the rate of surgery with instrumentation (88%) showed a strong trend toward posterior-instrumented fusion surgery in T-OPLL that is currently prevalent [5].

The previous study’s findings support IONM utilization in preventing postoperative motor palsy [6]. After IONM deterioration, rescue techniques that include pausing decompression, irrigation of the surgical area with warm saline, raising body temperature and blood pressure, completion of spinal cord decompression, and dekyphosis contribute to the intraoperative restoration of deteriorated IONM. Rescue procedures after IONM aggravation lessen postoperative motor palsy, although it is required to determine the appropriate alarm point for IONM in T-OPLL surgery and to design a treatment to prevent motor palsy after IONM aggravation [6].

In a prospective multicenter study by the monitoring committee of the Japanese Society for Spine Surgery and Related Research, patients with a transcranial motor-evoked potential (Tc-MEP) alert during posterior decompression and fusion surgery for T-OPLL were examined [6]. Tc-MEP alerts occurred during various procedures: decompression (60%), exposure (16%), rodding (6%), pedicle screw insertion (5%), posture change (5%), dekyphosis (3%), and other procedures (5%). After intraoperative intervention, the rescue rate was 57%, and rescue cases significantly improved preoperative ambulatory status and a much higher baseline waveform derivation rate [6]. These findings imply that intraoperative intervention following a Tc-MEP alert can effectively arrest the deterioration of postoperative neurological deficits [6].

## 2. Thoracic Ossification of the Ligamentum Flavum (T-OLF)

Although T-OLF is a relatively uncommon spinal condition globally, it is more prevalent in Asian nations. Due to the progressive disease and the poor response of conservative therapy, patients with T-OLF myelopathy generally require surgery. Notably, thermal nociception, improvement of vibration sensation disorder, history of trauma, and other concurrent ossifications of the spinal ligaments are associated factors regarding outcome-related aspects [7]. The presence of the discontinuous ossification of the anterior longitudinal ligament at the maximum stenosis, dural ossification, number of segments affected by OLF, symptom duration, and abnormal joint position sense of the hallux before surgery are also factors that correlate with postoperative outcomes [7]. However, no association exists between age, sex, symptom duration, diabetes and hypertension status, OLF levels, number of affected segments, or surgery type [7].

Preoperative Japanese Orthopedic Association for Myelopathy scores (JOA score) of less than 6 points, fused-type OLF, and beak-type OLF are factors related to surgical outcomes. Other factors include symptom duration, changes in intramedullary signal intensity on magnetic resonance imaging, longer duration of preoperative symptoms, and anteroposterior compression degree [7].

Whether decompression and fusion improve surgical results for OLF compared to decompression alone cannot be determined owing to data insufficiency. In the systematic review, randomized controlled trial (RCT), which directly compared the postoperative results of decompression alone with those of decompression and fusion for T-OLF, were not found. Notably, owing to the need for reoperation and poor improvement rate in symptom, postoperative kyphosis development can become problematic in some individuals when only laminectomy is used to treat T-OLF. Extensive laminectomy without fusion can minimize postoperative kyphosis progression without associated worsening symptoms, with noticeable postoperative alterations absent in thoracic kyphosis [7].

Patients who received laminectomy to remove the ossified lesion concomitant with lateral fusion utilizing bone graft alone experienced a considerably higher rate of JOA score improvement than those without simultaneous fusion. However, regardless of whether fusion was performed, no statistically significant difference was found between the preoperative and postoperative local kyphotic angles. Only a few studies in the same report compared decompression alone with decompression with fusion; however, the patients who underwent internal fusion were limited to those in whom the cervicothoracic junction or thoracolumbar junction was affected and those with osteoporosis [7].

The use of instrumentation can result in higher expenses and decreased spinal mobility due to decompression and fusion. However, some benefits include a reduction in the local kyphotic angle’s postoperative progression and an improvement in neurological symptoms. Therefore, it is not yet possible to conclude that simultaneous fusion considerably improves postoperative results. In addition, patients may find it challenging to select a treatment course given the hazy cost-effectiveness [7].

For patients who have instability before surgery and for those who need to have the facet joint removed for decompression and who are at risk for postoperative instability, simultaneous fusion may be the best option. However, additional studies are required to support these conclusions. It should be highlighted that the pathophysiology of OLF positioned posterior to the spinal cord fundamentally differs from that of OPLL located anterior to the spinal cord, and direct decompression can be performed for OLF via posterior decompression [7].

A multi-institutional retrospective study of patients receiving T-OLF surgery was conducted. The duration of symptoms and the presence of an ossified dura mater were significant predictors of surgical success [8]. A prospective countrywide multicenter study involving 223 T-OLF patients was conducted [9]. The rate of surgery with instrumentation (48.9%) illustrated the current strong trend toward posterior instrumentation during fusion surgery for T-OLF. Patients with severe OLF may benefit from fusion surgery with instrumentation. The optimal surgical timing and techniques for T-OLF should be the focus of additional prospective studies on long-term results to significantly enhance outcomes [9].

OLF occurs frequently at lower thoracic levels due to certain factors: increased mechanical stress caused by the thoracic spine’s position at the intersection of the lumbar spine’s elasticity and rib cage’s rigidity, a direct link between increased spinal mobility and recurrent mild trauma, and the posterior column’s high tensile force. Three cases of thoracic spinal cord injury without major bone injury associated with OLF were reported [10]. Furthermore, following injury by a low-energy method, spinal cord injury without significant bone injury of the lower thoracic spine should be suspected in geriatric populations, especially in patients with OLF. How to treat cervical spinal cord injuries without significant bone damage is being debated, as is the best treatment course for thoracic spinal cord injuries. Reducing neurological impairments and preventing their progression are two of the main objectives of treatment for spinal cord injuries. Patients with OLF in the thoracic spine who have had spinal cord injury but no significant bone damage may experience worsening symptoms and less favorable results. Decompression surgery should therefore be considered to prevent subsequent injuries. Due to the compressive etiology in the previous study, decompression surgery was performed on all patients with T-OLF, and postoperative neurological symptoms improved [10].

While the prospective evaluation of surgical treatment outcomes is made possible by focusing on perioperative complications and risk variables, the RCT of surgical procedures remains uniquely challenging. Spinal surgeons must continue to study these cases prospectively for an extended period to assess the surgical results over time and select the best surgical approach based on patient needs in the future.
